# Shared Genetics of Psychiatric Disorders

**DOI:** 10.12688/f1000research.18130.1

**Published:** 2019-09-12

**Authors:** Tova Fuller, Victor Reus

**Affiliations:** 1 Deptartment of Psychiatry, UCSF Weill Institute for Neurosciences, University of California, San Francisco School of Medicine, San Francisco, CA, USA

**Keywords:** psychiatry, transdiagnostic, genetics, genomics, polygenic, risk

## Abstract

Until recently, advances in understanding the genetic architecture of psychiatric disorders have been impeded by a historic, and often mandated, commitment to the use of traditional, and unvalidated, categorical diagnoses in isolation as the relevant phenotype. Such studies typically required lengthy structured interviews to delineate differences in the character and duration of behavioral symptomatology amongst disorders that were thought to be etiologic, and they were often underpowered as a result. Increasing acceptance of the fact that co-morbidity in psychiatric disorders is the rule rather than the exception has led to alternative designs in which shared dimensional symptomatology is analyzed as a quantitative trait and to association analyses in which combined polygenic risk scores are computationally compared across multiple traditional categorical diagnoses to identify both distinct and unique genetic and environmental elements. Increasing evidence that most mental disorders share many common genetic risk variants and environmental risk modifiers suggests that the broad spectrum of psychiatric pathology represents the pleiotropic display of a more limited series of pathologic events in neuronal development than was originally believed, regulated by many common risk variants and a smaller number of rare ones.

## Introduction

Investigation of the underlying genetic risk of individual mental disorders has been hampered by a number of factors, including a lack of validity for current categorical diagnoses and a high rate of co-morbidity amongst disorders, as well as the operational difficulty in accruing a sufficient number of subjects satisfying rigidly defined diagnostic requirements. In recent years, investigators have recognized that what was initially perceived as a problem may in fact represent a strength and that alternative approaches, focusing on genetic analysis of shared intermediate risk phenotypes in quantitative traits, such as cognition, dimensional symptomatology, and cortical structure and function, may represent a more direct probe of causality ^1– 3^. Such approaches have in turn engendered new statistical methods of analysis ^4– 6^. To this end, Plana-Ripoll *et al*. ^7^, in a large-scale study encompassing almost 84 million person years, have convincingly shown that co-morbidity is present across all psychiatric disorders and is bi-directional, meaning that the risk for any given additional mental disorder is increased by the presence of the first. Seemingly disparate psychiatric disorders then are likely to share common genetic risk variants and, depending on gene dosage and the additional contribution of rare variants and environmental modulation, represent pleiotropic versions of a pathology network that is more shared than distinct.

## Shared individual variants

One method of identifying overlap between disorders is to find individual variants shared by two or more disorders. A genome-wide association study (GWAS) of individuals with autism spectrum disorder (ASD) found a significant overlap with schizophrenia (SCZ) at 8p11.23, 3p25.3 ( *ATP2B2)*, and 3p13 ( *FOXP1)* ^8^. However, while the authors collected around 16,500 cases and approximately 16,000 controls, they did not find individual variants exceeding their appropriate GWAS threshold in the discovery set.

Similarly, in an older study, McCarthy *et al*. found copy number variation (CNV) duplications of 16p11.2 associated with SCZ as well as bipolar disorder (BPD) and depression ^9^. At the same time, the microduplications were rare, and the power to detect significance was low.

Xiao *et al*. ^10^ used prior research identifying two single nucleotide polymorphisms (SNPs), rs2709370 and rs6785, in the cAMP responsive element-binding (CREB)-1 gene to study associations with BPD, major depressive disorder (MDD), and SCZ. A meta-analysis found both SNPs were associated with increased risk of BPD ( *p* = 2.33 × 10 ^-4^ and 6.33 × 10 ^-5^, respectively). Likewise, an association with SCZ (3.96 × 10 ^-5^ and 2.44 × 10 ^-5^) and MDD ( *p* = 0.0144 and 0.0314) were identified. A number of studies demonstrating similar genetic overlap between other combinations of mental, medical, and neurologic disorders have also recently appeared ^11– 23^.

## Determining overlap using polygenic summary statistics

Since each individual locus has a small contribution to disease risk in complex disorders, creating corresponding issues with power that hinder identification, efforts have focused on polygenic attempts to determine shared risk. The methods covered below to estimate overlap in genetic loading include linkage disequilibrium score regression (LDSR) and polygenic risk scores (PRS) ^24^.

LDSR utilizes GWAS data to determine a linkage equilibrium score, defined as the sum of LD *r* ^2^ with all other SNPs, as well as to find heritability estimates and determine genetic correlations with different phenotypes. The common variant heritability ( *h* ^2^ *g*) of each disorder, or the proportion of phenotypic variance, is potentially explainable by an optimal linear predictor formed using additive effects of autosomal SNPs. The optimal predictors for two phenotypes can be correlated ^25^. The LDSR approach has limitations. If LD scores in the reference population approximate those in the target population, this will increase measurement error of the LDSR. Another issue of bias arises if LD scores in the reference population are increased or decreased with respect to those of the target population ^26^.

A PRS may, for example, prune and threshold associating statistics from a GWAS, then choose the best polygenic score based on a set of candidate polygenic scores for each disease. The GWAS is performed in the discovery sample with a PRS assigned to individuals for a given disorder (risk alleles weighted by their odds ratios). The coefficient of determination from regression analysis, R ^2^, is pruned by significance thresholds (P_T). The best polygenic score is often based on the maximal AUC. There are also limitations for the PRS method: the discovery sample must be as large as the original sample and both must be of significant size. The phenotype should be homogeneous (a problem in such disorders as MDD), and the level of genetic variation explained by common variants must be high as well ^27^.

As an example of these methods, one GWAS on alcohol dependence found genetic correlations based on LDSR with depressive symptoms, a diagnosis of MDD, attention deficit hyperactivity disorder (ADHD), SCZ, neuroticism, and subjective well-being at the *p* = 10 ^-5^ level or better ^28^. Likewise, a study using a summary statistic to determine polygenic risk correlated among 24 disorders found a strong overlap between SCZ and BPD, both of which were associated with depression ^29^. Similar associations have been reported for ADHD and eating disorders ^30^ and between substance abuse and psychotic disorders ^31, 32^.

In children, a PRS was used to determine the shared contribution among ADHD, several pediatric psychiatric disorders, depression, panic disorder, and generalized anxiety disorder (GAD) ^33^. In sum, Brikell and colleagues identified a general psychopathology factor suggesting a vulnerability to multiple disorders in children. Another study used PRS to determine overlap between SCZ and ASD with social communication difficulties at 8 and 17 years old ^34^.

A similar polygenic approach was employed by Selzam *et al*. ^35^ to posit a shared dimension which contributes to multiple disorders. A principal components analysis found a general “p” factor on which all disorders loaded that explained up to 60% of the variance, with SCZ, BPD, and depression the highest-loading disorders.

A large study by The Brainstorm Consortium (265,218 cases and 784,643 controls) published results from a GWAS demonstrating similarities among different psychiatric disorders and, in contrast, demonstrating differences with neurological disorders, except migraine ^25^. This study determined the common variant heritability of 17 disorders including multiple psychiatric disorders from disparate domains such as affective disorders, anxiety disorders, SCZ, ADHD, post-traumatic stress disorder (PTSD), and ASD. Findings demonstrated SCZ was genetically correlated with most of the other psychiatric disorders (average genetic correlation [ *r*_g_] = 0.40), while MDD was correlated with all. Clinically, these results are consistent with shared treatment guidelines for both MDD and anxiety disorders, shared symptomatology of depression with PTSD, and co-existence of mood disorders and SCZ in schizoaffective disorder. The aforementioned findings with migraine included an association between migraine and ADHD ( *r*_g_ = 0.26, *p* = 8.81 × 10 ^-8^), migraine and Tourette Syndrome ( *r*_g_ = 0.19, *p* = 1.80 × 10 ^-5^), and migraine and MDD ( *r*_g_ = 0.32, *p* = 1.42 × 10 ^-22^).

The above findings regarding neurological disease and its overlap with psychiatric pathology were replicated in at least one GWAS determining genetic overlap between MDD and migraine ^36^. LDSR was also used to demonstrate a 14.3% genetic correlation between SCZ and amyotrophic lateral sclerosis (ALS), with PRS for SCZ explaining 0.12% of the phenotypic variance in ALS, corresponding to a modest odds ratio of 1.08–1.26 ^37^. While there is little shared heritability with other neurological diseases, depression has a PRS associated with those of heart failure and ischemic disease, consistent with known phenomena such as depression after myocardial infarction ^38^.

An alternative approach has been to look at subphenotypes, such as rapid cycling and presence of psychosis in both BPD and SCZ patients ^39– 41^. Smeland *et al*. ^14^ took a somewhat different approach, examining how both SCZ and BPD compared in association with genes linked to cognitive performance. A number of unique associations were found, with most for BPD predicting better performance and for SCZ worse performance.

## Gene networking

Analysis of gene networks has built on transcription data to identify correlated transcripts/genes that form modules. These modules built from different transcripts that correlate with one another over different individuals form putative pathways of genes working in concert. The benefit of using such a method is a dimensional reduction analysis; rather than looking at single genes, the module “eigengene”, which roughly approximates the first principal component, is used for a group of genes hypothesized to be functioning in concert based on a pattern of up- or down-regulation together. Such gene networking approaches have demonstrated shared and differential module expression in BPD, ASD, ADHD, alcoholism, depression, and SCZ ^39, 42– 44^. ****

Expression studies of SCZ using post-mortem brain samples have been limited in size, but in a study of 92 medicated and 29 antipsychotic-free SCZ patients and 118 healthy controls, hierarchical clustering of 5,000 preselected transcripts was used to find modules of genes. Two in particular were highly expressed in the brain, and a statistic, *k-*within, was used to find hub genes in one module specifically related to SCZ ^45^.

## Conclusions

Although recent advances in identifying shared risk architecture are exciting, the timeline for clinical translation remains opaque. The number of implicated common risk variants is large and continues to grow, their individual effect sizes remain small, and methods for determining which rare variants are causally related and which are merely incidental are limited, as is our understanding of the role of epigenetic factors. Articulation of relevant pathologic pathways and spatiotemporal characterization of altered expression in development awaits further research, making potential therapeutic interventions based on these findings even more distant in the future. The first dramatic effect is likely to be in diagnostic classification and in how we consider matching patients to treatments and predicting prognosis.

A limitation of many of these studies is the use of mostly European ancestry, making results difficult to extrapolate to other populations. Power also continues to be an issue in detecting more rare variants, specifically in GWAS studies looking to replicate individual SNP or CNV findings.

One area of psychopathology not yet explored is that of personality disorders; there is known overlap, for example, between borderline personality disorder and MDD, anxiety disorders, and PTSD, although large-scale investigation of the genetics of core personality constructs has thus far resulted in conflicting findings. It might also be useful to further characterize the contribution from environmental experiences shared across disorders through concomitant use of SNP, CNV, and transcriptomic data.

Could shared heritability lead to neuroanatomical correlates? One natural step in this regard is to examine transdiagnostic neuroanatomical similarities coinciding with shared genetics, as Gong *et al*. ^46^ did, finding that SCZ, MDD, PTSD, and obsessive–compulsive disorder shared greater gray matter volume in the putamen on MRI ( *P* <0.001), which correlated with severity of symptoms. Similarly, van der Meer *et al*. ^47^ were able to link SCZ-associated genes to specific changes in hippocampal subfield volumes that were distinct from those seen in Alzheimer’s disease. Recent reports from the ENIGMA Consortium lend additional support to this avenue of inquiry ^48, 49^.
